# Outcomes and Predictors of Treatment Response in Patients With Pure Lupus Membranous Nephropathy

**DOI:** 10.1016/j.ekir.2026.106678

**Published:** 2026-07-08

**Authors:** Kevin Chevalier, Romain Brousse, Alexandre Karras, Nathalie Costedoat-Chalumeau, Julien Dang, Maxence Tailliar, Noémie Jourde-Chiche, Charles Ronsin, Moglie Le Quintrec-Donnette, Marie Julien, Stanislas Faguer, Aymeric Couturier, Pierre Pommerolle, Céline Lebas, Antoine Coussement, Marion Chapal, Christophe Barba, Alexia Gauffre, Maxime Teisseyre, Johan Noble, Chloé Comarmond, Eve Vilaine, Jean-François Augusto, Aurélie Hummel, Évangéline Pillebout, Jean-Michel Halimi, Olivier Moranne, Eric Daugas, Jean-Jacques Boffa, Emmanuel Esteve, Lupus Nephritis French Cooperative Group

**Affiliations:** 1 Department of Nephrology, Tenon Hospital, Assistance Publique-Hôpitaux de Paris, Institut national de la santé et de la recherche médicale (INSERM) CORAKID U155, Sorbonne University, Paris, France; 2 Department of Nephrology, European Hospital Georges Pompidou, Assistance Publique-Hôpitaux de Paris, Université Paris Cité, Paris, France; 3 Department of Internal Medicine, Reference Center for Rare Systemic Auto Immune and Autoinflammatory Diseases of Ile de France East and West, Cochin Hospital, Assistance Publique-Hôpitaux de Paris, Université de Paris, Paris, France; 4 Department of Nephrology and Transplantation, Bicêtre Hospital, Assistance Publique des Hôpitaux de Paris (AP-HP), Université Paris-Saclay, Le Kremlin-Bicêtre, France; 5 Department of Nephrology and Renal Transplantation, Hôpital de la Conception, Assistance Publique-Hôpitaux de Marseille, Marseille, France; 6 Department of Nephrology and Immunology, Nantes University Hospital, Nantes, France; 7 Department of Nephrology, Intensive Care, Dialysis, and Transplantation, Hôpital Lapeyronie, Montpellier, France; 8 Nephrology Department, CHU de la Réunion, Saint Denis, France; 9 Département de Néphrologie et Transplantation d’Organes, Axe Transplantation – Immunité – Environnement, CHU de Toulouse, France; 10 Nephrology Unit, American Hospital of Paris, Neuilly-sur-Seine, France; 11 Department of Nephrology, Dialysis, and Transplantation, University of Picardie Jules Verne, Amiens University Hospital, Amiens, France; 12 Department of Nephrology and Renal Transplantation, Centre Hospitalier Régional Universitaire de Lille, Lille, France; 13 Department of Nephrology, CHU de Dijon, Dijon, France; 14 Department of Nephrology, Hospital of Vendée, La Roche-Sur-Yon, France; 15 Department of Nephrology and Nutrition, Hospices Civils de Lyon, Centre Hospitalier Lyon-Sud, Pierre- Bénite, France; 16 Department of Nephrology, CHU de Rouen, Rouen, France; 17 Département de Néphrologie, Dialyse et Transplantation, CHU de Nice, Université Côte d'Azur, France; 18 Department of Nephrology, Grenoble-Alpes University Hospital, Grenoble, France; 19 Internal Medicine Department, Département Médico-Universitaire INVICTUS, Lariboisière Hospital, Assistance Publique-Hôpitaux de Paris, Nord-Université Paris-Cité, Paris, France; 20 Department of Nephrology - Hemodialysis, CHU Ambroise Paré, Assistance Publique-Hôpitaux de Paris (AP-HP), Université Versailles-Saint Quentin-en-Yvelines, Boulogne-Billancourt, France; 21 Department of Nephrology-Dialysis-Transplantation, Université d'Angers, CHU Angers, Angers, France; 22 Department of Nephrology, Necker-Enfants Malades Hospital, Assistance Publique-Hôpitaux de Paris, Paris, France; 23 Department of Nephrology, Saint Louis Hospital, Paris, France; 24 Department of Nephrology, UMR1327 Ischemia, Investigation Network Initiative – Cardiovascular and Renal Clinical Trialists (INI-CRCT), Tours University Hospital, Tours University, Tours, France; 25 Department of Nephrology, Dialysis and Apheresis, CHU de Nîmes, IDESP Universite de Montpellier, France; 26 Nephrology Department, Assistance Publique-Hôpitaux de Paris, Bichat Hospital, Université Paris Cité and INSERM U1149, Paris, France

**Keywords:** class V lupus nephritis, membranous nephropathy, mycophenolate mofetil, rituximab, systemic lupus erythematosus

## Abstract

**Introduction:**

The indications and modalities of therapeutic strategies for first-onset pure lupus membranous nephropathy (PLMN) remain poorly defined. We aimed to evaluate renal response rates across real-world treatment strategies and identify predictors of treatment response in PLMN.

**Methods:**

We conducted a multicenter retrospective study in 25 French centers. Patients with biopsy-proven first-onset PLMN treated between 2000 and 2020 were included. Patients were classified according to their initial treatment strategy. Renal response rates were defined based on the 2024 Kidney Disease: Improving Global Outcomes (KDIGO) guidelines. Factors associated with treatment failure at 12 months were assessed using logistic regression.

**Results:**

Among 194 patients with PLMN, 53 (27.3%) received supportive care only, and 141 (72.7%) received immunosuppressive therapy (54 [38.3%] received mycophenolate mofetil (MMF)-based regimens, 26 [18.4%] received rituximab [RTX]-based regimens, and 61 [43.3%] received other treatments). At 12 months, rates of complete renal response (CRR) and CRR or partial renal (PRR) were respectively, 36.7% and 55.1% with supportive care, 47.1% and 60.8% with MMF, 64.0% and 72.0% with RTX, and 45.4% and 54.5% with other regimens. In multivariable analysis, the presence of anti–U1-ribonucleoprotein (U1RNP) antibodies was independently associated with treatment failure (adjusted odds ratio [OR]: 2.53; 95% confidence interval [CI]: 1.35–4.70; *P* = 0.004), whereas extrarenal lupus involvement was protective (adjusted OR = 0.39; 95% CI: 0.19–0.73; *P* = 0.004). RTX-based regimens were significantly associated with shorter treatment duration and corticosteroid sparing.

**Conclusion:**

In patients with first-onset PLMN, 12-month renal response rates did not differ significantly among immunosuppressive regimens despite variation in treatment duration and steroid exposure. Anti-U1RNP antibodies and absence of extrarenal involvement were associated with a higher risk of treatment failure. Anti-CD20–based strategies may be a promising therapeutic approach.

## Graphical abstract

**Figure:**
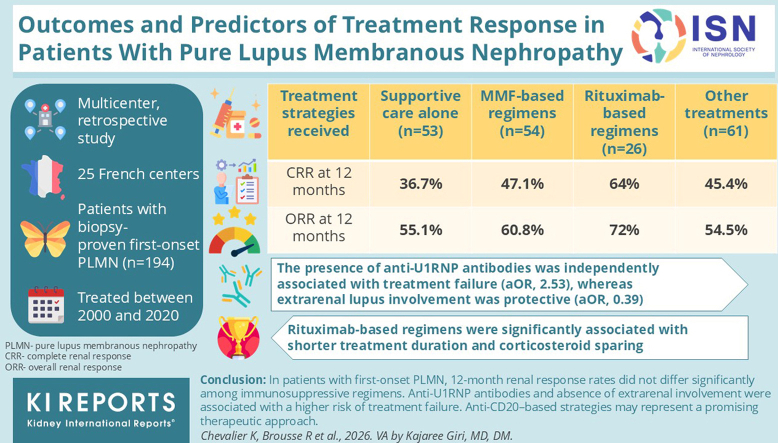

Renal involvement affects ≤60% of patients with systemic lupus erythematosus (SLE) and constitutes a major prognostic factor associated with decreased health-related quality of life and increased mortality.^1^, ^2^, ^3^, ^4^ Proliferative forms of lupus nephritis (LN), namely ISN / Renal Pathology Society classification classes IIIA and IVA, represent the most common and severe forms of LN.^5^^,^^6^ As such, they have gathered significant attention and effort, resulting in high grade international consensus to guide therapeutic decisions and follow-up.^1^^,^^7^, ^8^, ^9^, ^10^, ^11^ PLMN, corresponding to class V LN, accounts for 5% to 20% of biopsy-proven LN cases.^12^ Although this class is associated with lower mortality and a less severe prognosis than proliferative ones,^13^^,^^14^ kidney failure still occurs in 10% to 30% of affected patients.^15^ This supports the addition of antimalarials and immunosuppressive agents to the usual supportive care that consists of antiproteinuric agents, and lifestyle measures in the most severe cases. Nevertheless, indications and modalities of such therapy remain debated. Most guidelines recommend initiating immunosuppression based on persistent or high-range proteinuria, but thresholds vary from 1 to 3 g/d.^7^, ^8^, ^9^, ^10^, ^11^ The 2024 KDIGO guidelines do not favor any specific regimen among MMF, cyclophosphamide, calcineurin inhibitors (CNI), RTX, or azathioprine.^7^ In the same year, the American College of Rheumatology (ACR) issued updated recommendations advocating a more intensive approach, proposing glucocorticoid (GC)-based combination therapy (GC + MMF + CNI) for patients with proteinuria > 1 g/g, and GC combined with MMF, azathioprine, or CNI for less severe presentations, while reserving anti-CD20 therapy for refractory cases only.^11^ Most data on kidney outcomes in these patients come from small trials (16–60 patients), pooled series (≤75 cases), or observational studies (13–38 patients).^16^, ^17^, ^18^, ^19^, ^20^, ^21^, ^22^, ^23^, ^24^, ^25^ Furthermore, these regimens were originally developed for proliferative LN and may not be well-suited to the distinct context of PLMN.^26^, ^27^, ^28^

To address these gaps and provide evidence in an unselected setting, we conducted a nationwide, multicenter, retrospective investigation to evaluate renal response rates across current immunosuppressive strategies for first-onset PLMN and to identify the predictors of treatment success.

## Methods

### Study Design

We performed a retrospective, observational, nationwide study focusing on patients diagnosed with a first flare of PLMN between 2000 and 2020, involving 25 tertiary centers in France. Consecutive patients were screened from local databases and/or from patients’ records in pathology departments. Eligible patients were aged ≥18 years with a diagnosis of SLE according to the ACR/ European Alliance of Associations for Rheumatology 2019 criteria^29^ with a first-onset biopsy-proven PLMN according to the 2003 ISN/ Renal Pathology Society classification.^30^ Patients and the public were not involved in the design, conduct, reporting, or dissemination plans of this research.

### Data Collection

Data were collected using REDCap electronic data capture tools hosted at Assistance Publique-Hôpitaux de Paris.^31^^,^^32^ Recorded variables included sex, ethnicity, weight, body mass index, age at PLNM diagnosis, clinical and biological characteristics of SLE, kidney biopsy characteristics, treatments, and their associated adverse events. Sex was self-reported by participants at inclusion. In this work the term “sex” is used to denote self-reported sex, without distinction between biological sex and gender identity. Data were collected at diagnosis, at 6 months, 12 months, and at last known follow-up.

### Definitions

Baseline was defined as the date of PLNM diagnosis on kidney biopsy, and follow-up was defined as the time between baseline and the last available visit. Nephrotic syndrome was defined as urine protein-to-creatinine ratio (UPCR) >3 g/g and/or urine albumin-to-creatinine ratio >3 g/g with serum albumin <30 g/l. Renal responses were defined according to the 2024 KDIGO guidelines.^7^ Thus, complete renal response (CRR) was defined as an UPCR <0.5 g/g with a stabilization (decrease <15% of baseline estimated glomerular filtration rate [eGFR]) or an improvement in kidney function. Partial renal response (PRR) was defined as a >50% reduction in proteinuria from baseline, with a UPCR between 0.5 and 3 g/g and stabilization (decrease <15% of baseline eGFR) or improvement in kidney function. Absence of kidney response was defined as not reaching PRR or CRR. eGFR was computed using the Chronic Kidney Disease–Epidemiology Collaboration 2021 race-free creatinine-based equation for all patients.^33^

Four different groups of treatment were determined based on the initial therapy received. The 4 groups are as follows: (i) the supportive care group, receiving only angiotensin-converting enzyme inhibitors, angiotensin II receptor blockers, and/or sodium-glucose cotransporter 2 inhibitor, without any disease-modifying antirheumatic drugs or immunosuppressive therapy (including corticosteroids ≥20 mg/d of prednisone equivalent); (ii) the MMF group, receiving MMF with low- or high-dose corticosteroids; (iii) the RTX group receiving RTX (either 375 mg/m^2^/wk × 4 or 1 g on day 0 and day 14) with low or high dose corticosteroids; and (iv) the other treatments group of patients treated with other immunosuppressive drugs, including corticosteroids alone ≥ 20 mg/d, cyclophosphamide, azathioprine, methotrexate, belimumab, CNI or a combination of ≥2 immunosuppressive treatments (Supplementary Figure S1). High-dose corticosteroids were defined as a prednisone-equivalent starting dose ≥ 20 mg/d, and low-dose corticosteroids as <20 mg/d, in accordance with European Alliance of Associations for Rheumatology / European Renal Association–European Dialysis and Transplant Association recommendations.^34^ Only the prednisone-equivalent starting dose of long-term oral corticosteroid therapy was taken into account. I.V. corticosteroid pulse doses, including those administered as premedication for RTX infusions, were not included in corticosteroid exposure assessment. Antimalarial prescription was assessed in the 4 groups but did not contribute to the treatment group definition. Treatment failure was defined as the absence of PRR or CRR at 12 months, the occurrence of a relapse before 12 months, or a change in therapeutic class not considered maintenance therapy by the prescribing physician.

Because treatment changes occurring early after diagnosis may introduce bias in retrospective analyses, we additionally performed a sensitivity analysis inspired by target trial emulation principles, in which patients were reassigned according to the treatment received within the first 3 months after treatment initiation. Patients who switched therapy within this time were analyzed according to the treatment received at month 3, irrespective of their initial assignment.

Characteristics of patients experiencing an early treatment change and the corresponding treatment reclassification are presented in Supplementary Table S1, and renal outcomes according to this alternative assignment strategy are reported in Supplementary Table S2.

### Ethical Approval

This study complied with the Declaration of Helsinki. According to French regulation, every patient was duly informed and consented to the use of their personal data. The Foch Hospital Ethical Review committee (registration number IRB00012437) approval was given under the reference 24-03-06.

### Statistical Analyses

Continuous variables were described using means and SDs or medians and interquartile ranges. We compared distributions between 2 independent groups using the Mann–Whitney U test and proportions using the chi-square test or Fisher exact test as appropriate. Comparison of continuous variables in >2 independent groups was performed using the Kruskal–Wallis test.

The association between clinical, histological, and immunological variables and treatment failure was assessed using univariable then multivariable logistic regression in STATA (version 17, StataCorp). The multivariable model was obtained by entering risk factors that met Wald's test *P* ≤ 0.20 in univariable analyses; covariates included in the final multivariable model were identified using stepwise backward elimination until each covariate was independently associated with treatment failure with a *P*-value < 0.05. To address confounding by indication in the comparison of treatment strategies, additional multivariable logistic regression analyses were performed with renal response at 12 months as the outcome and RTX-based regimens as the exposure of interest, adjusting for baseline variables that differed between treatment groups and are plausible determinants of treatment response: baseline proteinuria (log-transformed), initial prednisone-equivalent dose, hydroxychloroquine use, and Afro-Caribbean ancestry. These analyses were conducted for 2 outcomes (CRR and CRR+PRR) and 3 comparators (MMF-based regimens, other immunosuppressive regimens, and all non-RTX regimens combined), and are presented as exploratory because of the limited number of events per variable.

Survival analyses were performed in R (version 4.6.1) using the survival (v3.8.3) and ggsurvfit (v1.2.0) packages.^35^ Survival without renal or extrarenal flares was estimated using the Kaplan–Meier method, with administrative censoring at the date of last known visit. Group comparisons were evaluated using the log-rank test and pairwise comparisons with Bonferroni correction. For visual clarity, survival curves are truncated at 62.5 months, corresponding to the time point at which <10% of patients remained at risk in at least one treatment group.^36^

Changes in proteinuria, serum albumin, and steroid dose (prednisone equivalent/d) between day 0, month 6, and month 12 were analyzed using linear mixed-effects models that included time, treatment group, and their interaction as fixed effects, with subject as a random effect, using the lme4 (v1.1.37) and lmerTest (v3.1.3) R packages; *P*-values for fixed effects were obtained through Satterthwaite's approximation. Missing data were not replaced. Two-tailed *P*-values < 0.05 were considered statistically significant.

## Results

### Patient Characteristics at Baseline

A total of 218 patients with a first PLMN flare were identified. Among them, 1 patient withdrew his consent to participate in the study and 23 patients had experienced ≥1 previous flares of mixed class LN (III + V or IV + V) before inclusion. Finally, 194 patients with a first PLMN flare were included for analysis (Supplementary Figure S2). The median (interquartile range) follow-up was 5.4 (3.1–8.4) years and the median time between lupus diagnosis and PLMN diagnosis was 1.5 (0–7.8) years.

Baseline characteristics are presented in detail in Table 1. Briefly, most patients were female (*n* = 170, 87.6%) with a median age of 28 (21–36) years at SLE diagnosis. Eighty-six patients (44.3%) were of Afro-Caribbean origin. Thirty-eight patients (19.6%) had experienced a previous non–class V renal lupus flare. Among them, 28 of 38 (73.7%) had had class III or IV LN. The median time between previous renal flare and the diagnosis of PLMN was 36.2 (11.2–58.7) months.

**Table 1:** Patients’ characteristics at lupus diagnosis and at baseline (pure class V lupus nephritis diagnosis)

Characteristics	Number	Values
Demographic		
Age at lupus diagnostic, yr – median (IQR)	194	27.5 (21–36)
Female sex, *n* (%)	194	170 (87.6)
African-Caribbean ancestry, *n* (%)	194	86 (44.3)
Prior manifestations		
Prior lupus flare, *n* (%)	194	114 (58.8)
Isolated renal flare		3 (2.2)
Extrarenal flare		76 (66.7)
Combined renal and extrarenal flare		35 (30.7)
ISN-RPS 2003 previous kidney flare class	194	38 (100)
I		3 (7.9)
II		7 (18.4)
III		7 (18.4)
IV		21 (55)
Time between previous biopsy and current LMN flare (mo) – median IQR	38	36.2 (11.2–58.7)
Characteristics at PLMN diagnosis		
Age, yr – median (IQR)	194	33.9 (26.4–40.0)
eGFR (CKD-EPI 2021 race-free creatinine-base) – ml/min per 1.73 m^2^ media (IQR)	194	122.1 (98.7–133.6)
Serum albumin – g/l median (IQR)	181	28.6 (21–33)
UPCR – g/g median (IQR)	188	2.3 (1.1–4.4)
Hematuria, – *n* (%)	176	76 (43.2)
C3 – mg/l, median (IQR)	151	820 (580–1080)
C4 – mg/l, median (IQR)	147	140 (80–230)
Antinuclear antibody with titer ≥ 1:80 – *n* (%)	194	194 (100)
Positive anti-dsDNA Abs – *n* (%)	194	130 (67.0)
Positive anti-Smith Abs – *n* (%)	194	74 (38.1)
Positive anti-U1RNP Abs – *n* (%)	194	85 (43.8)
Positive anti-SSA Abs – *n* (%)	194	72 (37.1)
Positive anti-SSB Abs – *n* (%)	194	19 (9.8)
Arthralgia – *n* (%)	194	64 (33.3)
Cutaneous involvement – *n* (%)	194	63 (32.5)
Leucopenia – *n* (%)	189	36 (19.0)
Thrombocytopenia – *n* (%)	189	17 (9.0)
Absence of extrarenal involvement – *n* (%)	194	64 (33.0)
Histopathologic features at PLMN diagnosis		
Percentage of obsolescent glomeruli median (IQR)	171	0 (0–9.1)
Interstitial fibrosis – tubular atrophy	170	
Absent (<10%) – *n* (%)		137 (80.6)
Mild (≥ 10% to < 25 %) – *n* (%)		23 (13.5)
Moderate (≥ 25% to 50 %) – *n* (%)		4 (2.4)
Severe (≥ 50% to 100 %) – *n* (%)		6 (3.5)
IgG membranous deposits by IF – *n* (%)	166	166 (100)
Glomerular deposits		
IgA deposits – *n* (%)	167	87 (52.1)
IgM deposits – *n* (%)	167	88 (52.7)
C3 deposits – *n* (%)	167	148 (88.6)
C1q deposits – *n* (%)	167	130 (77.8)
Full-house deposits – *n* (%)	167	57 (34.1)
Follow-up		
Follow-up – yrs, median (IQR)	178	5.43 (3.12–8.43)

Abs, antibodies; CKD-EPI, Chronic Kidney Disease–Epidemiology Collaboration equation; dsDNA, double strain DNA; eGFR, estimated glomerular filtration rate; IF, immunofluorescence; IQR, interquartile range; ISN/RPS: International Society of Nephrology/Renal Pathology Society; LMN, lupus membranous nephropathy; LN, lupus nephritis; PLMN, pure LMN; Sm, Smith; U1RNP, U1 ribonucleoprotein; UPCR, urine protein creatinine ratio.

For 7 patients, the age at lupus diagnosis could not be precisely determined from the available electronic medical records. For these patients, age at lupus diagnosis was imputed using age at diagnosis of pure lupus membranous nephropathy.

Number: Number of participants remaining after accounting for missing data.

At diagnosis of PLMN, the median 2021 Chronic Kidney Disease–Epidemiology Collaboration eGFR was 122.1 (98.7–133.6) ml/min per 1.73 m^2^, serum albumin was 28.6 (21–33) g/l, and median UPCR was 2.2 (1.2–4.5) g/g. A total of 67 of 180 patients (37.2%) had nephrotic syndrome. One hundred thirty patients (67.0%) had extrarenal lupus manifestations, summarized in Table 1.

### Therapeutic Regimens and Outcomes

Of the 194 patients, 53 (27.3%) received only supportive care, and 141 (72.7%) were treated with ≥1 immunosuppressive agents (Table 2). Consistent with international guidelines, patients receiving immunosuppression had higher UPCR levels (*P* < 0.01), lower serum albumin (*P* < 0.001), and were more likely to present with nephrotic syndrome (*P* < 0.01).

**Table 2:** Treatments of class V LN and their efficacy

Characteristics and outcomes	Supportive care only (*n* = 53)	Immunosuppressive therapy (*n* = 141)	*P*-value	MMF-based therapy (*n* = 54)	Rituximab-based therapy (*n* = 26)	Other therapies (*n* = 61)	*P*-value
Prior class III or IV LN flare	7/53 (13.2)	21/141 (14.9)	0.77	7/54 (13.0)	6/26 (23.1)	8/61 (13.1)	0.43
Hydroxychloroquine – *n* (%)	41/51 (80.4)	111/139 (79.9)	0.93	45/54 (83.3)	24/26 (92.3)	42/59 (71.2)	0.06
Therapeutic switch during the first 24 mo – *n* (%)	10/53 (18.9)	32/141 (22.7)	0.56	9/54 (16.7)	2/26 (7.7)	21/61 (34.4)	0.01
Treatment duration (mo), median, (IQR)	31.3 (12.5–85.1)	11.9 (4.3–32.6)	< 0.001	25.3 (7.3–36.3)	6.2 (0.9–19.4)	8.0 (3.3–38.1)	< 0.01
Prednisone equivalent starting dose—mg/d, median (IQR)	0 (0 – 5)	35 (10 – 60)	< 0.001	40 (20 – 60)	10 (0 – 60)	40 (20 – 60)	0.02
UPCr < 1 g/g, *n* (%)	13/51 (25.5)	26/137 (19.0)	0.33	6/53 (11.3)	5/26 (19.2)	15/58 (25.9)	0.15
UPCr ≥ 1 to ≤ 3 g/g, *n* (%)	28/51 (54.9)	46/137 (33.6)	< 0.01	24/53 (45.3)	6/26 (23.1)	16/58 (27.6)	0.07
UPCr > 3 g/g, *n* (%)	10/51 (19.6)	65/137 (47.4)	< 0.001	23/53 (43.4)	15/26 (57.7)	27/58 (47.5)	0.48
Nephrotic syndrome – *n* (%)	9/51 (17.6)	57/129 (44.2)	< 0.01	23/51 (45.1)	15/24 (62.5)	24/54 (44.4)	0.29
eGFR (CKD-EPI), median (IQR)	123.2 (100.0 – 130.2)	121.6 (97.7 – 135.5)	0.64	118.8 (94.7 – 136.8)	119.6 (93.9 – 134.2)	124.3 (104.4 – 135.5)	0.91
PRR at 6 mos	6/47 (12.8)	24/124 (19.3)	0.31	12/50 (24.0)	8/23 (34.8)	4/51 (7.8)	0.01
CRR at 6 mos	1/47 (2.1)	30/124 (24.2)	< 0.01	11/50 (22)	5/23 (21.7)	14/51 (27.4)	0.78
ORR at 6 mos	7/47 (14.9)	54/124 (43.5)	< 0.001	23/50 (46.0)	13/23 (56.5)	18/51 (35.3)	0.21
PRR at 12 mos	9/49 (18.4)	14/131 (10.7)	0.17	7/51 (13.7)	2/25 (8.0)	5/55 (9.1)	0.66
CRR at 12 mos	18/49 (36.7)	65/131 (49.6)	0.12	24/51 (47.1)	16/25 (64)	25/55 (45.4)	0.27
ORR at 12 mos	27/49 (55.1)	79/131 (60.3)	0.53	31/51 (60.8)	18/25 (72)	30/55 (54.5)	0.33
Class V LN relapse during follow up – *n* (%)	12/49 (24.5)	22/137 (16.1)	0.19	9/52 (17.3)	3/25 (12.0)	11/60 (18.3)	0.82
Proliferative class (III or IV) renal relapse during follow up – *n* (%)	7/53 (13.2)	13/141 (9.2)	0.42	6/54 (11.1)	2/26 (7.7)	5/61 (8.2)	0.83
Extra-renal relapse during follow up – *n* (%)	12/53 (22.6)	30/141 (21.3)	0.84	11/54 (20.4)	4/26 (15.4)	15/61 (24.6)	0.62
Follow up duration – yrs median (IQR)	6.33 (3.42–9.75)	5.19 (3.01–8.13)	0.31	5.06 (2.94–8.28)	3.03 (2.3–5.67)	5.93 (3.82–8.47)	0.03

Abs, antibodies; CKD-EPI, Chronic Kidney Disease–Epidemiology Collaboration equation; CRR, complete renal response; eGFR, estimated glomerular filtration rate; IQR, interquartile range; LN, lupus nephritis, ORR, overall renal response, PRR, partial renal response; UPCR, urine protein creatinine ratio.

Accordingly, among patients initially managed with supportive care only, most had low to moderate baseline proteinuria. Specifically, 13 of 51 (25.5%) patients had proteinuria <1 g/g, 28 of 51 (54.9%) had proteinuria between 1 and 3 g/g, and 10 of 51 (19.6%) had proteinuria > 3 g/g at treatment initiation (Table 2). Outcomes of patients treated with supportive care only stratified by baseline proteinuria are presented in detail in Supplementary Table S3. Among the 10 patients with baseline proteinuria >3 g/g, none achieved PRR or CRR at 6 or 12 months, and 4 of 10 (40%) required initiation of immunosuppressive therapy for inefficacy within the first 12 months.

**Figure 1:**
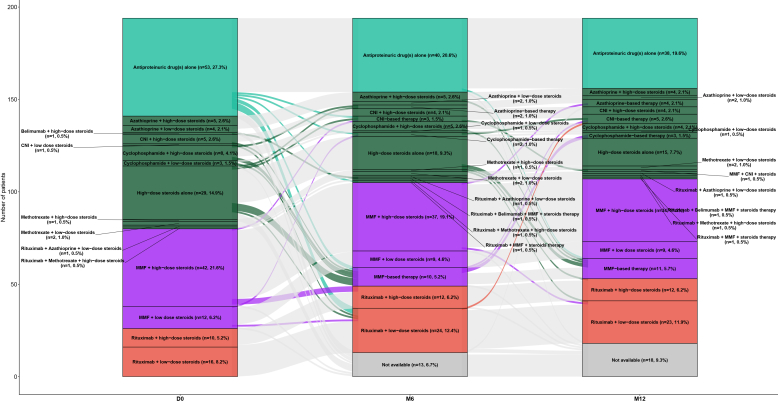
Sankey plot showing the evolution of treatment at 6 and 12 months of follow-up. Sankey diagram illustrating treatment patterns at D0, M6, and M12. Each stratum represents a treatment regimen, with height proportional to the number of patients. Stratum colors indicate therapeutic groups: supportive care only (turquoise); other immunosuppressive regimens including CNIs (green); MMF ± steroids (purple); rituximab ± steroids (red); treatment not available (light gray). Flows represent treatment trajectories over time. Gray flows indicate patients remaining on the same therapeutic class (including steroid dose adjustment only), whereas colored flows indicate treatment switches, with the color corresponding to the initial treatment group. Numbers in parentheses indicate patient counts and percentages at each time point. CNI, calcineurin inhibitor; D0, baseline; M6, month 6, M12, month 12; MMF, mycophenolate mofetil.

**Figure 2:**
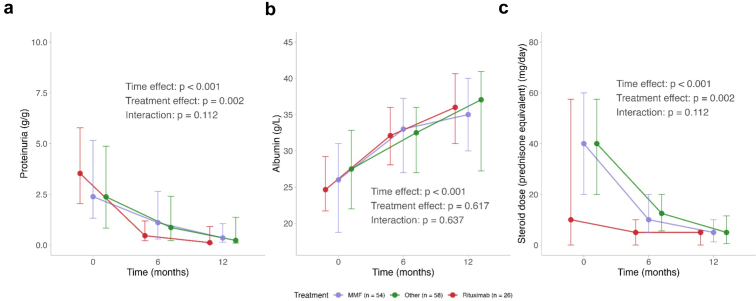
Evolution of (a) proteinuria (urine protein-to-creatinine ratio), (b) serum albumin, and (c) corticosteroid dosage at month 0, 6, and 12 according to the initial immunosuppressant group of treatment. Panels show median values of (a) urinary protein-to-creatinine ratio, (b) serum albumin, and (c) prednisone-equivalent steroid dose at day 0, month 6, and month 12, by treatment group. Error bars represent interquartile ranges (Q1–Q3). *P*-values were derived from linear mixed-effects models, including time, treatment group, and their interaction as fixed effects, with patient as a random effect.

Within the 141 patients (72.7%) who received an immunosuppressive drug as first line therapy, 54 (38.3%), 26 (18.4%), and 61 (43.2%) were treated by MMF-based regimen, RTX-based regimen, or another regimen, respectively (Table 2). Details of RTX therapeutic regimens are summarized in Supplementary Table S4 and the evolution of drug prescription over time during the first 12 months are in Figure 1. During the initial 12-month follow-up, treatment was switched in 10 patients (18.9%) receiving supportive care, 8 (14.8%) in the MMF group, 1 (3.8%) in the RTX group, and 18 (31.0%) in the other treatments group (*P* = 0.02). As per definition, these patients were subsequently analyzed as treatment failures. Treatment duration differed significantly across the 4 groups (*P* < 0.01), with the shortest median duration observed in the RTX group. Proteinuria significantly decreased over time between treatment initiation (day 0) and months 6 and 12 in all groups (*P* < 0.001). A linear mixed-effects model, including time and treatment group did not show significant differences between groups (*P* = 0.45), or a significant time-by-treatment interaction (*P* = 0.26), indicating similar trajectories of proteinuria reduction across immunosuppressive regimens over 12 months. Accordingly, serum albumin levels increased significantly over time (*P* < 0.001), with no significant differences between treatment groups (*P* = 0.62) or time-by-treatment interaction (*P* = 0.64). Although corticosteroid doses differed significantly between treatment groups (*P* = 0.002), likely because of lower corticosteroid use in the RTX group, all patients experienced a decrease over the initial 12 months of treatment (*P* < 0.001). No significant time-by-treatment interaction was observed (*P* = 0.11), suggesting similar tapering patterns across groups (Figure 2).

By month 6, only 1 of 47 patients (2.1%) in the supportive care group achieved CRR, compared with 30 of 124 patients (24.2%) in the immunosuppressive groups (*P* < 0.01). These 30 responders were evenly distributed across treatment groups: 11 of 50 (22%) in the MMF group, 5 of 23 (21.7%) in the RTX group, and 14 of 51 (27.4%) in the other treatments group (*P* = 0.78).

By month 12, the complete response rate increased to 18 of 49 (36.7%) in the supportive care group and 65 of 131 (49.6%) in the immunosuppressive group (*P* = 0.12), with no significant difference between regimens: 24 of 51 (47.1%) in the MMF group, 16 of 25 (64.0%) in the RTX group, and 25 of 55 (45.4%) in the other treatments group (*P* = 0.27). Overall response rate, including both PRR and CRR at month 12, was comparable: 27 of 49 (55.1%) in the supportive care group versus 79 of 131 (60.3%) in the immunosuppressive group (*P* = 0.53), with no significant differences observed between immunosuppressive strategies (*P* = 0.33) (Table 2). Reassigning patients according to the treatment received within the first 3 months did not significantly change renal response rates at 6 or 12 months across treatment groups (Supplementary Tables S1 and S2). Given the nonrandomized design and the potential for confounding by indication, exploratory adjusted logistic regression analyses were performed to compare RTX-based regimens with MMF-based regimens, other immunosuppressive regimens, and all non-RTX immunosuppressive regimens combined. Models were adjusted for baseline proteinuria, initial prednisone-equivalent dose, hydroxychloroquine use, and Afro-Caribbean ancestry. Two outcomes were assessed at 12 months: CRR and overall renal response, defined as CRR or PRR. Across these models, RTX-based regimens were associated with numerically higher odds of renal response, with adjusted ORs ranging from 2.13 to 2.33 for CRR and from 1.77 to 2.25 for overall renal response. However, because of the low number of events per group, CIs were wide and none of these associations reached statistical significance (*P* = 0.11–0.32; Supplementary Table S5).

**Figure 3:**
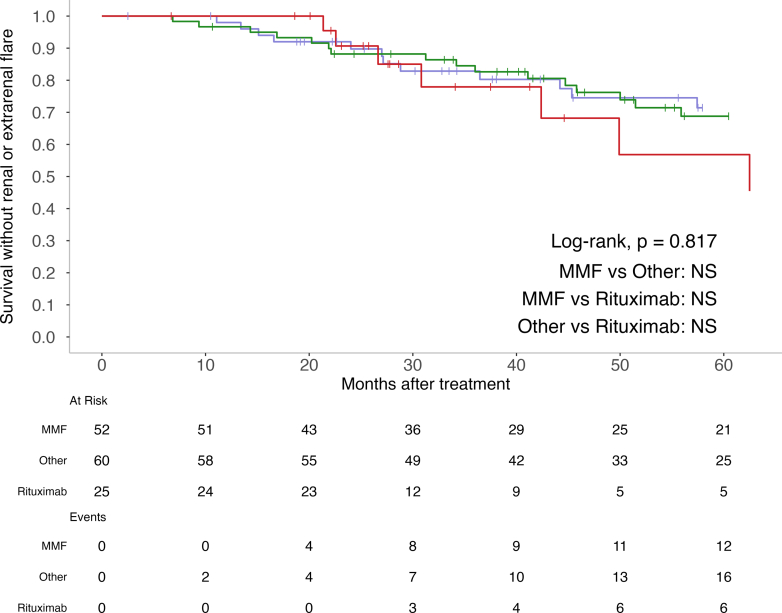
Relapse-free survival from renal or extrarenal flare. Shown is the probability of survival free from renal or extrarenal flare, as assessed by Kaplan-Meier estimation, among patients with pure lupus membranous nephropathy receiving immunosuppressive therapy, according to their initial treatment regimen. Numbers at risk and cumulative events are shown below the figure. *P*-values for pairwise comparisons were adjusted using the Bonferroni method.

The probability of renal and/or extrarenal flare did not differ significantly among the different treatment groups over the available follow-up period (Figure 3, Log-rank test, *P* = 0.817).There was no difference in the proportion of PLMN relapses (12/49 [24.5%] for supportive care group, 9/52 [17.3%] for MMF-based regimen group, 3/25 [12.0%] for RTX-based regimen group, and 11/60 [18.3%] for other immunosuppressant groups, *P* = 0.6). After a median follow-up of 5.4 [3.1-8.4] years, 9 patients reached kidney failure requiring kidney replacement therapy. This included 2 patients in the supportive care group (1 hemodialysis, 1 transplant), 3 in the MMF group (2 hemodialysis, 1 transplant), and 5 among those receiving other therapies (4 hemodialysis, 1 preemptive transplant). No patients in the RTX group required kidney replacement therapy; however, their follow-up was shorter.

### Risk of Treatment Failure

Associations between clinical, functional, immunological factors, and treatment failure are presented in Table 3. In univariable analysis, no significant association was found with demographic variables, proteinuria level, eGFR, or steroid dose. By contrast, the presence of anti-U1RNP antibodies was associated with higher odds of treatment failure (OR = 2.5; 95% CI: 1.3–4.5; *P* = 0.004), whereas extrarenal involvement was associated with a lower risk (OR = 0.39; 95% CI: 0.2–0.7; *P* = 0.004). Both associations remained significant in multivariable analysis after stepwise backward elimination (anti-U1RNP: OR = 2.5; 95% CI: 1.4–4.7; *P* = 0.004; extrarenal involvement: OR = 0.4; 95% CI: 0.2–0.7; *P* = 0.004). Of note, the seroprevalence of anti-U1RNP antibodies did not differ significantly between patients with and those without extrarenal involvement (43.1% vs. 45.3%, *P* = 0.77). After additional adjustment for first-line treatment strategy (immunosuppressive therapy vs. supportive care only), both anti-U1RNP positivity (OR = 2.46; 95% CI: 1.31–4.61; *P* = 0.005) and extrarenal involvement (OR = 0.40; 95% CI: 0.21–0.78; *P* = 0.007) remained independently associated with treatment failure. Baseline proteinuria level (log-transformed) was not significantly associated with treatment failure, either in univariable analysis (OR = 1.21; 95% CI: 0.88–1.67; *P* = 0.24) or after adjustment for treatment strategy (OR = 1.26; 95% CI: 0.90–1.74; *P* = 0.17).

**Table 3:** Independent determinants of treatment failure: univariable and multivariable analysis

Variable				Univariable analysis			Multvariable analysis		
		Number of patients	Number of treatment failure at 12 mos	OR	95% CI	*p*	OR	95% CI	*p*
Age (/1-yr increment)		180	73	0.992	0.966–1.019	0.548			
Sex	Male	23	10	1					
	Female	157	63	0.871	0.360–2.110	0.760			
Origin	Non–Afro-Caribbean ancestry	101	40	1					
	Afro-Caribbean ancestry	79	33	1.094	0.601–1.992	0.769			
Previous renal flare	No	144	56	1					
	Yes	36	17	1.406	0.674–2.933	0.364			
Time between lupus and PLMN diagnoses (/1-yr increment)		173	80	0.987	0.946–1.030	0.562			
CKD-EPI (per 1 ml/min per 1/73 m^2^ increment)		180	73	0.999	0.989–1.009	0.839			
Proteinuria (continuous) (per 1 g/g increment)		176	72	1.175	0.854–1.616	0.322			
Nephrotic syndrome	No	108	43	1					
	Yes	62	28	1.245	0.622–2.340	0.497			
Urinary protein-to-creatinine ratio	≤ 1 g/g	140	14	1		-			
	> 1 to < 3 g/g	36	26	0.739	0.381–1.981	-			
	≥ 3 g/g	103	33	1.525	0.669–3.476	0.249			
Extrarenal involvement	No	59	33	1			1		
	Yes	121	40	0.389	0.205–0.737	0.004	0.377	0.194–0.728	0.004
C3 levels at diagnosis (/1 mg/l increment)		141	57	0.688	0.257–1.848	0.459			
Presence of anti-dsDNA	No	57	24	1					
	Yes	123	49	0.910	0.481–1.723	0.773			
Presence of anti-Sm	No	112	44	1					
	Yes	68	29	1.149	0.623–2.120	0.656			
Presence of anti-U1RNP	No	100	31	1			1		
	Yes	80	42	2.460	1.337–4.528	0.004	2.534	1.353–4.747	0.004
Presence of anti-SSA	No	114	46	1					
	Yes	66	27	1.023	0.552–1.896	0.941			
Presence of anti-SSB	No	161	65	1					
	Yes	19	8	1.074	0.410–2.815	0.884			
Dose of corticosteroids (/1 mg increment)		180	74	0.993	0.981–1.006	0.423			
High dose (≥ 20 mg/d) of corticosteroids	No	84	38	1					
	Yes	96	36	0.726	0.400–1.318	0.293			
Hydroxychloroquine prescription	No	35	18	1					
	Yes	143	54	0.573	0.272–1.206	0.142			

Abs, antibodies; CI, confidence interval; dsDNA, double strain DNA, eGFR, estimated glomerular filtration rate; OR, odds ratio; Sm, Smith; U1 RNP, U1 ribonucleoprotein.

The final multivariable logistic regression model was obtained by entering the risk factors from the univariable models that met *P* ≤ 0.20 as the threshold in a single multivariable logistic regression model. Covariates included in the multivariate model were extrarenal involvement, presence of anti-U1RNP and hydroxychloroquine prescription. After using a stepwise backward elimination, the final multivariate model identified each covariate associated with treatment failure with a value < 0.05. Association was performed using the Wald’s test.

When comparing patients with and those without anti-U1RNP antibodies, those with anti-U1RNP were more frequently of Afro-Caribbean ancestry (*P* < 0.001), more often positive for anti-Sm antibodies (*P* < 0.001), and had less prior lupus kidney involvement (*P* = 0.005). Compared with patients without anti-U1RNP antibodies, these patients received a higher initial dose of GCs (*P* < 0.001), were more frequently treated with MMF (*P* = 0.001), and less frequently treated with RTX (*P* = 0.007) (Supplementary Table S6).

Patients with and those without extrarenal involvement were largely comparable, except that the interval between lupus diagnosis and PLMN was longer in patients without extrarenal involvement (*P* < 0.01), and prior lupus kidney involvement was more frequent in this group (*P* =0 .01) (Supplementary Table S7).

## Discussion

To our knowledge, this study represents the largest cohort of unselected patients with biopsy-proven first-onset PLMN, featuring validated end points, extended follow-up, and comprehensive clinical and therapeutic phenotyping. Our main findings are as follows: (i) supportive care only may be a viable option in carefully selected patients; (ii) as in idiopathic membranous nephropathy, the trajectory of proteinuria is typically slow, suggesting that the absence of improvement at month 6, although proposed by KDIGO and ACR as a potential indicator of treatment failure, should be interpreted with caution; and (3) we confirm the potential efficacy of low-dose corticosteroid and anti-CD20–based regimens.

Compared with previously reported cohorts, participants in our study had lower UPCR, higher serum albumin levels, and were less likely to present with nephrotic syndrome or progress to kidney failure at last follow-up (*n* = 10, 4.6%, compared with 10%–30% reported in the literature).^7^^,^^16^^,^^21^, ^22^, ^23^^,^^37^ In addition, we report a higher proportion of patients with Afro-Caribbean ancestry. These differences likely reflect the real-world nature of our cohort, which had no proteinuria-based inclusion criteria, as well as specific demographic characteristics in France and evolving biopsy thresholds and surveillance practices in patients with SLE. Nevertheless, the renal response rates we report are in accordance with previous series.^17^^,^^19^^,^^21^, ^22^, ^23^, ^24^^,^^38^

We identified >26 distinct initial treatment regimens (Supplementary Figure S1) before consolidating them into 4 groups, underscoring the lack of consensus regarding immunosuppressive treatment in PLMN. As expected and consistent with current guidelines, patients receiving supportive care only presented with lower baseline proteinuria and higher serum albumin levels than those treated with immunosuppressive agents. Importantly, this apparent efficacy of supportive care should be interpreted in the context of patient selection: nearly 80% of patients managed with supportive care only had baseline proteinuria <3 g/g, whereas outcomes were poor among the small subgroup with high-range proteinuria. Although renal response rates were significantly lower in the supportive care group at 6 months, this difference was no longer observed at 12 months, suggesting that supportive care may represent an appropriate initial strategy in a highly selected, lower-risk subgroup, highlighting that conditional immunosuppressive treatment recommended by the 2024 ACR guidelines may not be uniformly required in this population, which is in line with the more conservative approach of by the 2024 KDIGO recommendations.^7^^,^^11^ Among the immunosuppressive agents prescribed by French physicians for the treatment of PLMN, MMF and RTX were the most commonly used regimens, with overall prescription rates (including first and subsequent lines of therapy) of 73 of 194 (37.6%) and 40 of 194 (20.6%), respectively. Only 15 of 194 patients (7.7%) were exposed to cyclophosphamide, a former cornerstone in the standard of care for LN. This limited use may reflect concerns regarding its less favorable safety profile, its association with premature gonadal failure,^26^^,^^27^ and potential long-term malignancy risk.^28^ The reduced-dose cyclophosphamide regimen evaluated in the EUROLUPUS trial may help mitigate these adverse effects.^39^^,^^40^ Compared with other reported cohorts, CNIs were less frequently prescribed in our study (13/194, 6.7%), which might be related to their known association with metabolic and vascular complications, higher relapse rates after discontinuation,^17^ and the burden of nephrotoxicity requiring close therapeutic monitoring. The growing body of evidence supporting next-generation CNIs, such as voclosporin, with improved efficacy and safety profiles, may significantly reshape this therapeutic landscape in the near future.^41^^,^^42^

Azathioprine, shown to be less effective in maintaining renal response in proliferative LN^43^ was only prescribed to 14 of 194 patients (7.2%). Although only few small studies have specifically investigated MMF in PLMN,^16^^,^^18^^,^^21^ its effectiveness in proliferative LN and well-established favorable safety profile^7^, ^8^, ^9^, ^10^ likely contributed to its popularity in our cohort.

There is a growing body of evidence supporting the use of anti-CD20 therapies in LN, and PLMN may represent a particularly suitable indication. RTX use in this setting is supported by a prior multicenter retrospective study in which 14 of 15 patients with PLMN achieved a PRR or CRR,^23^ and possibly by the RITUXILUP study, which evaluated RTX and MMF without corticosteroids in LN and included a substantial proportion of patients with PLMN (22/50, 44%).^44^ More recently, other B-cell–targeted therapies such as obinutuzumab^45^^,^^46^ and belimumab^47^ have shown promising results in LN, and anti-CD19 chimeric antigen receptor T-cell therapy demonstrated high efficacy in refractory cases.^48^ The increased use of RTX in clinical practice may reflect its expanding role in non–lupus membranous nephropathy^49^, ^50^, ^51^ and its inclusion as a therapeutic option for PLMN in the 2021 KDIGO guidelines. More recently, among the subgroups analyzed in the REGENCY trial, patients with concomitant class V LN appeared to derive the greatest benefit from the addition of obinutuzumab to standard therapy.^46^ In our study, RTX-based regimens were associated with comparable unadjusted renal response and flare rates compared with other immunosuppressive strategies, despite shorter treatment duration and lower corticosteroid exposure. However, direct comparisons between treatment regimens should be interpreted with caution, because of the retrospective design and the likelihood of confounding by indication. To partly address this issue, we performed exploratory adjusted analyses accounting for baseline differences between treatment groups. Although these models consistently showed numerically higher odds of renal response with RTX-based regimens, the estimates were imprecise and did not reach statistical significance. These findings therefore support RTX-based regimens as a promising steroid-sparing strategy in PLMN; however, that should not be interpreted as evidence of superior efficacy. Prospective studies are needed to better define the role of B-cell–depleting therapies in this setting. Current therapeutic decisions in PLMN heavily rely on eGFR and proteinuria, but our study reveals the limitations of this approach. We found that neither marker at baseline was associated with treatment response. Moreover, their inherently slow evolution makes them poorly suited for timely therapeutic adjustments, especially given the wide variety of disease presentations. This is clearly underscored by the significant increase in remission rates observed between 6 and 12 months in many patients, even without therapy modification.

These observations underscore an urgent need for more dynamic, longitudinal assessment and the integration of superior, and more importantly, precocious prognostic markers.

In our multivariable analysis, anti-U1RNP antibody positivity was independently associated with treatment failure. Although primarily a hallmark of mixed connective tissue disease, these antibodies are also found in SLE, where they modulate clinical phenotype.^52^, ^53^, ^54^ Notably, several cohorts have reported a higher prevalence of anti-U1RNP antibodies in patients with membranous (pure or mixed class V) LN compared with proliferative forms.^55^^,^^56^ Recent evidence links U1RNP antibodies to renal involvement in systemic sclerosis via endothelial injury.^57^ Mechanistically, anti-U1RNP antibodies may induce endothelial inflammation and dysfunction through direct binding and upregulation of adhesion molecules.^58^^,^^59^ These findings are consistent with the emerging concept that endothelial-podocyte crosstalk plays a critical role in the pathogenesis of proteinuric kidney diseases, as recently illustrated by the contribution of endothelial-derived adhesion molecules such as CD93 to podocyte injury.^60^ Importantly, we carefully explored potential confounding factors and did not identify any clinical, immunological, or therapeutic variable that accounted for the observed association. Nevertheless, we cannot exclude that anti-U1RNP antibodies may, at least in part, represent a surrogate marker of heightened immune activity rather than a direct pathogenic driver. Disentangling whether anti-U1RNP antibodies act as mediators of glomerular injury or merely reflect a more aggressive immunological milieu will require dedicated mechanistic and longitudinal studies.

Conversely, extrarenal manifestations were associated with better renal outcomes. This unexpected finding should be interpreted with caution and should not be considered evidence of a causal protective effect of extrarenal involvement. Patients with extrarenal manifestations differed from those without extrarenal involvement in several respects, including a shorter interval between lupus diagnosis and kidney biopsy, a lower frequency of prior renal involvement, and a trend toward higher initial GC exposure. These differences may suggest that these patients were assessed at an earlier or clinically different phase of the disease, possibly because overt extrarenal manifestations prompted earlier diagnostic evaluation and treatment initiation. However, this interpretation is only partial, because age, baseline eGFR, proteinuria, and serum albumin levels were similar across groups. Alternative explanations, including differences in health care engagement, disease awareness, or treatment adherence among patients with clinically overt systemic disease, may have contributed, although these hypotheses remain speculative. Overall, these findings should be viewed as hypothesis-generating and require confirmation in larger cohorts.^61^

Despite being the largest reported cohort of biopsy-proven PLMN, our study has limitations. As with any retrospective design, referral bias and incomplete case capture cannot be excluded. The absence of centralized pathology review limited our ability to assess emerging histological markers, such as exostosin-1 or -2. In addition, the cohort reflects French clinical practice and population characteristics, potentially limiting generalizability. The retrospective design limited our ability to draw conclusions regarding treatment indications; to define optimal proteinuria thresholds for initiating immunosuppression, if such thresholds exist; or to make robust comparisons of efficacy across treatment regimens, because unmeasured confounding factors may have influenced initial treatment decisions and introduced bias. Furthermore, despite the overall cohort size, the individual treatment groups were small, which may have resulted in insufficient statistical power and thus a lack of detectable differences. In addition, the number of events within each group was too limited to allow for robust multivariable modeling (e.g., multinomial logistic regression). Although GC exposure was included in the analyses, heterogeneity in its use across treatment strategies limited assessment of its independent effect on renal outcomes. Furthermore, i.v. corticosteroid pulse doses administered during the induction phase were not systematically captured and could not be included in the analyses. This represents a potential source of residual confounding, particularly given that pulse i.v. steroids may have contributed differentially to early treatment responses across groups. Exposure to renin-angiotensin system blockade was not specifically collected; however, angiotensin-converting enzyme inhibitor or angiotensin receptor blocker therapy was expected to be prescribed to the majority of patients according to contemporaneous recommendations, limiting the relevance of assessing its independent effect on renal outcomes. Because the inclusion period was restricted to patients diagnosed before 2020, only a small number were exposed to sodium-glucose cotransporter 2 inhibitors or belimumab, precluding any meaningful analysis of the effects of these therapies. Similarly, triple-agent (multitarget) immunosuppressive regimens, as advocated in the most recent ACR recommendations, were almost never used in this cohort, with treatment strategies largely limited to monotherapy or dual-agent combinations.^11^ Nonetheless, the study’s strengths include its large sample size, real-world design, long-term follow-up, and granular, patient-level data encompassing clinical, histological, immunological, and therapeutic domains.

In conclusion, CRR at 12 months was achieved in 36.7% of patients who initially received supportive care only, 47.1% of those treated with MMF, 64.0% of those receiving RTX, and 45.4% of those treated with another immunosuppressive regimen. The rates of CRR or PRR were 55.1% in the supportive care group, 60.8% in the MMF group, 72.0% in the RTX group, and 54.5% in the group receiving other immunosuppressive therapies. Despite similar trajectories of proteinuria and serum albumin, and comparable rates of renal and extrarenal flare during follow-up across groups, RTX was associated with reduced corticosteroid exposure without compromising remission rates, supporting its prospective evaluation, along with other B-cell–targeting therapies. Treatment failure was not associated with baseline proteinuria levels or steroid dose, but rather with the presence of anti-U1RNP antibodies and the absence of extrarenal manifestations, challenging proteinuria-centered treatment paradigms and underscoring the need for more individualized strategies in PLMN management.

## Appendix

### List of the Lupus Nephritis French Cooperative Group

Jean-François Augusto, Christelle Barbet, Julie Bellière, Ramia Benhamou, Eléonore Bettacchioli, Mickaël Bobot, Jean-Jacques Boffa, Sebastien Boutinet, Romain Brousse, Valérie Caudwell, Agnès Chapelet, Clara Chapez, Nicolas Charles, Kevin Chevalier, Gabriel Choukroun, Camille Cohen, Lionel Couzi, Nathalie Costedoat-Chalumeau, Etienne Crickx, Eric Daugas, Stan Faguer, Victor Gueutin, Matthieu Halfon, Catherine Harotel Saliou, Julien Hogan, Alice Horisberger, Pierre Housset, Estel-Anaïs Hubaud, Aurélie Hummel, Nizar Joher, Anne Jolivot, Noémie Jourde Chiche, Alexandre Karras, Ludivine Lebourg, Emmanuel Ledoult, Bénédicte Levy, Grégoire Martin de Frémont, Alexis Mathian, Olivier Moranne, Bruno Moulin, Clovis Mugnier, Laeticia Normand, Marine Novion, Julie Oniszczuk, Solenne Pelletier, Evangeline Pillebout, Thomas Quemeneur, Karim Sacré, Aurélie Sannier, Quentin Simon, Ludovic Tréfond.

## Disclosure

KC reported receiving a research fellowship grant from the Institut National de la Santé et de la Recherche Médicale (National Institute of Health and Medical Research). RB reported receiving a research fellowship grant from the Fondation pour la Recherche Médicale (French Foundation for Medical Research). All the other authors declared no competing interests.

## Data Availability Statement

The data supporting the findings of this study and the statistical codes used can be supplied on request in the event of a reasonable request.

### Declaration of AI and AI-Assisted Technologies in the Writing Process

During the preparation of this work, the authors used ChatGPT 4.o in order to improve English readability. After using this tool/service, the authors reviewed and edited the content as needed and take full responsibility for the content of the publication.

## Supplementary Material

Supplementary File (PDF) **Figure S1.** Sankey plot showing group allocation by number of patients. **Figure S2.** Flow chart. **Table S1.** Characteristics of patients who switched therapy within the first 3 months after treatment initiation. **Table S2.** Sensitivity analysis of renal outcomes after reassignment of patients according to treatment received within the first 3 months. **Table S3.** Renal outcomes of patients treated with supportive care only according to baseline proteinuria. **Table S4.** Details of rituximab therapeutic regimens. **Table S5.** Multivariate logic regression analyses comparing renal response rates between rituximab-based regimens and other treatments groups. **Table S6.** Comparison between anti-U1RNP positive and anti-U1RNP negative patients. **Table S7.** Comparison between patients with or without extrarenal involvement.

## Footnotes

Supplementary File (PDF)
**Figure S1.** Sankey plot showing group allocation by number of patients. **Figure S2.** Flow chart. **Table S1.** Characteristics of patients who switched therapy within the first 3 months after treatment initiation. **Table S2.** Sensitivity analysis of renal outcomes after reassignment of patients according to treatment received within the first 3 months. **Table S3.** Renal outcomes of patients treated with supportive care only according to baseline proteinuria. **Table S4.** Details of rituximab therapeutic regimens. **Table S5.** Multivariate logic regression analyses comparing renal response rates between rituximab-based regimens and other treatments groups. **Table S6.** Comparison between anti-U1RNP positive and anti-U1RNP negative patients. **Table S7.** Comparison between patients with or without extrarenal involvement.

## References

[1] Anders H.J., Saxena R., Zhao M.H., Parodis I., Salmon J.E., Mohan C. (2020). Lupus nephritis. Nat Rev Dis Primers.

[2] Cervera R., Khamashta M.A., Font J. (2003). Morbidity and mortality in systemic lupus erythematosus during a 10-year period: a comparison of early and late manifestations in a cohort of 1,000 patients. Medicine.

[3] Kaul A., Gordon C., Crow M.K. (2016). Systemic lupus erythematosus. Nat Rev Dis Primers.

[4] Mejia-Vilet J.M., Turner-Stokes T., Houssiau F., Rovin B.H. (2023). Kidney involvement in systemic lupus erythematosus: from the patient assessment to a tailored treatment. Best Pract Res Clin Rheumatol.

[5] Weening J.J., D’Agati V.D., Schwartz M.M. (2004). The classification of glomerulonephritis in systemic lupus erythematosus revisited. Kidney Int.

[6] Bajema I.M., Wilhelmus S., Alpers C.E. (2018). Revision of the International Society of Nephrology/Renal Pathology Society classification for lupus nephritis: clarification of definitions, and modified National Institutes of Health activity and chronicity indices. Kidney Int.

[7] Kidney Disease: Improving Global Outcomes (KDIGO) Lupus Nephritis Work Group (2024). KDIGO 2024 Clinical Practice Guideline for the management of LUPUS NEPHRITIS. Kidney Int.

[8] Fanouriakis A., Kostopoulou M., Andersen J. (2024). EULAR recommendations for the management of systemic lupus erythematosus: 2023 update. Ann Rheum Dis.

[9] Mok C.C., Hamijoyo L., Kasitanon N. (2021). The Asia Pacific League of Associations for Rheumatology consensus statements on the management of systemic lupus erythematosus. Lancet Rheumatol.

[10] Haute Autorité de Santé (HAS) (2024). Lupus Systémique de l’adulte et de l’enfant. https://www.has-sante.fr/jcms/p_3493410/fr/lupus-systemique-de-l-adulte-et-de-l-enfant.

[11] Sammaritano L.R., Askanase A., Bermas B.L. (2025). 2024 American College of Rheumatology (ACR) guideline for the screening, treatment, and management of lupus nephritis. Arthritis Rheumatol.

[12] Mok C.C. (2009). Membranous nephropathy in systemic lupus erythematosus: a therapeutic enigma. Nat Rev Nephrol.

[13] Tektonidou M.G., Dasgupta A., Ward M.M. (2016). Risk of end-stage renal disease in patients with lupus nephritis, 1971–2015: a systematic review and bayesian meta-analysis. Arthritis Rheumatol.

[14] Mok C.C., Kwok R.C.L., Yip P.S.F. (2013). Effect of renal disease on the standardized mortality ratio and life expectancy of patients with systemic lupus erythematosus. Arthritis Rheum.

[15] Mercadal L., Montcel S.T., Nochy D. (2002). Factors affecting outcome and prognosis in membranous lupus nephropathy. Nephrol Dial Transplant.

[16] Radhakrishnan J., Moutzouris D.A., Ginzler E.M., Solomons N., Siempos I.I., Appel G.B. (2010). Mycophenolate mofetil and intravenous cyclophosphamide are similar as induction therapy for class V lupus nephritis. Kidney Int.

[17] Austin H.A., Illei G.G., Braun M.J., Balow J.E. (2009). Randomized, controlled trial of prednisone, cyclophosphamide, and cyclosporine in lupus membranous nephropathy. JASN.

[18] Cramer C.H., Mills M., Valentini R.P., Smoyer W.E., Haftel H., Brophy P.D. (2007). Clinical presentation and outcome in a cohort of paediatric patients with membranous lupus nephritis. Nephrol Dial Transplant.

[19] Mok C.C., Ying K.Y., Lau C.S. (2004). Treatment of pure membranous lupus nephropathy with prednisone and azathioprine: an open-label trial. Am J Kidney Dis.

[20] Mok C.C., Ying K.Y., Yim C.W., Ng W.L., Wong W.S. (2009). Very long-term outcome of pure lupus membranous nephropathy treated with glucocorticoid and azathioprine. Lupus.

[21] Spetie D.N., Tang Y., Rovin B.H. (2004). Mycophenolate therapy of SLE membranous nephropathy. Kidney Int.

[22] Szeto C.C., Kwan B.C.H., Lai F.M.M. (2008). Tacrolimus for the treatment of systemic lupus erythematosus with pure class V nephritis. Rheumatology (Oxford).

[23] Chavarot N., Verhelst D., Pardon A. (2017). Rituximab alone as induction therapy for membranous lupus nephritis: a multicenter retrospective study. Medicine.

[24] Arriens C., Teng Y.K.O., Ginzler E.M. (2023). Update on the efficacy and safety profile of voclosporin: an integrated analysis of clinical trials in lupus nephritis. Arthritis Care Res.

[25] Mejía-Vilet J.M., Córdova-Sánchez B.M., Uribe-Uribe N.O., Correa-Rotter R. (2016). Immunosuppressive treatment for pure membranous lupus nephropathy in a Hispanic population. Clin Rheumatol.

[26] Chemaitilly W., Li Z., Krasin M.J. (2017). Premature ovarian insufficiency in childhood cancer survivors: a report from the St. Jude lifetime cohort. J Clin Endocrinol Metab.

[27] Boumpas D.T., Austin H.A., Vaughan E.M., Yarboro C.H., Klippel J.H., Balow J.E. (1993). Risk for sustained amenorrhea in patients with systemic lupus erythematosus receiving intermittent pulse cyclophosphamide therapy. Ann Intern Med.

[28] Van Den Brand J.A.J.G., Van Dijk P.R., Hofstra J.M., Wetzels J.F.M. (2014). Cancer risk after cyclophosphamide treatment in idiopathic membranous nephropathy. Clin J Am Soc Nephrol.

[29] Aringer M., Costenbader K., Daikh D. (2019). 2019 European League Against Rheumatism/American College of Rheumatology classification criteria for systemic lupus erythematosus. Ann Rheum Dis.

[30] Weening J.J., D’Agati V.D., Schwartz M.M. (2004). The classification of glomerulonephritis in systemic lupus erythematosus revisited. J Am Soc Nephrol.

[31] Harris P.A., Taylor R., Minor B.L. (2019). The REDCap consortium: building an international community of software platform partners. J Biomed Inform.

[32] Harris P.A., Taylor R., Thielke R., Payne J., Gonzalez N., Conde J.G. (2009). Research Electronic Data Capture (REDCap)—a metadata-driven methodology and workflow process for providing translational research informatics support. J Biomed Inform.

[33] Inker L.A., Eneanya N.D., Coresh J. (2021). New creatinine- and cystatin C–based equations to estimate GFR without race. N Engl J Med.

[34] Fanouriakis A., Kostopoulou M., Alunno A. (2019). 2019 update of the EULAR recommendations for the management of systemic lupus erythematosus. Ann Rheum Dis.

[35] R Core Team (2025). R: A Language and Environment for Statistical Computing.

[36] Pocock S.J., Clayton T.C., Altman D.G. (2002). Survival plots of time-to-event outcomes in clinical trials: good practice and pitfalls. Lancet.

[37] Yap D.Y., Yu X., Chen X. (2012). Pilot 24 month study to compare mycophenolate mofetil and tacrolimus in the treatment of membranous lupus nephritis with nephrotic syndrome. Nephrology (Carlton).

[38] Saïdi M., Brochériou I., Estève E. (2021). The exostosin immunohistochemical status differentiates lupus membranous nephropathy subsets with different outcomes. Kidney Int Rep.

[39] Tamirou F., Husson S.N., Gruson D., Debiève F., Lauwerys B.R., Houssiau F.A. (2017). Brief report: the euro-lupus low-dose intravenous cyclophosphamide regimen does not impact the ovarian reserve, as measured by serum levels of anti-Müllerian hormone. Arthritis Rheumatol.

[40] Houssiau F.A., Vasconcelos C., D’Cruz D. (2010). The 10-year follow-up data of the Euro-Lupus Nephritis Trial comparing low dose and high-dose intravenous cyclophosphamide. Ann Rheum Dis.

[41] Rovin B.H., Teng Y.K.O., Ginzler E.M. (2021). Efficacy and safety of voclosporin versus placebo for lupus nephritis (Aurora 1): a double-blind, randomised, multicentre, placebo-controlled, phase 3 trial. Lancet.

[42] Saxena A., Ginzler E.M., Gibson K. (2024). Safety and efficacy of long-term voclosporin treatment for lupus nephritis in the Phase 3 Aurora 2 clinical trial. Arthritis Rheumatol.

[43] Dooley M.A., Jayne D., Ginzler E.M. (2011). Mycophenolate versus azathioprine as Maintenance Therapy for lupus Nephritis. N Engl J Med.

[44] Condon M.B., Ashby D., Pepper R.J. (2013). Prospective observational single-centre cohort study to evaluate the effectiveness of treating lupus nephritis with rituximab and mycophenolate mofetil but no oral steroids. Ann Rheum Dis.

[45] Furie R.A., Aroca G., Cascino M.D. (2022). B-cell depletion with obinutuzumab for the treatment of proliferative lupus nephritis: a randomised, double-blind, placebo-controlled trial. Ann Rheum Dis.

[46] Furie R.A., Rovin B.H., Garg J.P. (2025). Efficacy and safety of obinutuzumab in active lupus nephritis. N Engl J Med.

[47] Furie R., Rovin B.H., Houssiau F. (2020). Two-year, randomized, controlled trial of Belimumab in lupus nephritis. N Engl J Med.

[48] Müller F., Taubmann J., Bucci L. (2024). CD19 CAR T-cell therapy in autoimmune disease — A case series with follow-up. N Engl J Med.

[49] Praga M., Barrio V., Juárez G.F., Luño J. (2007). Members of the Group listed at the end of the paper). Tacrolimus monotherapy in membranous nephropathy: a randomized controlled trial. Kidney Int.

[50] Howman A., Chapman T.L., Langdon M.M. (2013). Immunosuppression for progressive membranous nephropathy: a UK randomised controlled trial. Lancet.

[51] Fervenza F.C., Appel G.B., Barbour S.J. (2019). Rituximab or cyclosporine in the treatment of membranous nephropathy. N Engl J Med.

[52] Dima A., Jurcut C., Baicus C. (2018). The impact of anti-U1-RNP positivity: systemic lupus erythematosus versus mixed connective tissue disease. Rheumatol Int.

[53] Ben Brahim M., Daada S., Olfa J. (2021). Significations cliniques des anticorps anti-RNP au cours du lupus érythémateux systémique. Rev Med Interne.

[54] Elhani I., Khoy K., Mariotte D. (2022). The diagnostic challenge of patients with anti-U1-RNP antibodies. Rheumatol Int.

[55] Farinha F., Pepper R.J., Oliveira D.G., McDonnell T., Isenberg D.A., Rahman A. (2020). Outcomes of membranous and proliferative lupus nephritis – analysis of a single-centre cohort with more than 30 years of follow-up. Rheumatology (Oxford).

[56] Farinha F., Barreira S., Couto M. (2025). Risk of chronic kidney disease in 260 patients with lupus nephritis: analysis of a nationwide multicentre cohort with up to 35 years of follow-up. Rheumatology (Oxford).

[57] Chevalier K., Chassagnon G., Leonard-Louis S. (2024). Anti-U1RNP antibodies are associated with a distinct clinical phenotype and a worse survival in patients with systemic sclerosis. J Autoimmun.

[58] Okawa-Takatsuji M., Aotsuka S., Fujinami M., Uwatoko S., Kinoshita M., Sumiya M. (2001). Up-regulation of intercellular adhesion molecule-1 (ICAM-1), endothelial leucocyte adhesion molecule-1 (ELAM-1) and class II MHC molecules on pulmonary artery endothelial cells by antibodies against U1-ribonucleoprotein. Clin Exp Immunol.

[59] Okawa-Takatsuji M., Aotsuka S., Uwatoko S. (2008). Endothelial cell-binding activity of anti-U1-ribonucleoprotein antibodies in patients with connective tissue diseases. Clin Exp Immunol.

[60] Bauer C., Piani F., Troost J. (2026). Endothelial cell–released CD93 contributes to podocyte injury in idiopathic nephrotic syndrome. Sci Transl Med.

[61] Oliveira-Santos M., Verani J.F.S., Klumb E.M., Albuquerque E.M.N. (2011). Evaluation of adherence to drug treatment in patients with systemic lupus erythematosus in Brazil. Lupus.

