# Supplementary material for: Outcomes and Predictors of Treatment Response in Patients With Pure Lupus Membranous Nephropathy
Source: Kidney Int Rep. 2026 Jul 8;11(9):106678. doi: 10.1016/j.ekir.2026.106678 (PMC13393404; doi:10.1016/j.ekir.2026.106678)

SUPPLEMENTARY FILES

Supplementary table S1 - Characteristics of patients who switched therapy within the first 3 months after treatment initiation.

Patient number	Time between treatment start and switch (months)	Original treatment	Subsequent treatment after switch	Treatment modality as analyzed in the original manuscript	Treatment modality as defined by the treatment received at 3 months from therapy beginning
1	1,25	MMF + high-dose steroids	Rituximab + low-dose steroids	MMF group	Rituximab group
2	1,45	Belimumab + high-dose steroids	Rituximab + belimumab + MMF + steroids	Other drugs group	Other drugs group
3	1,71	High-dose steroids alone	MMF + high-dose steroids	Other drugs group	MMF group
4	2,20	Antiproteinuric drug(s) alone	Rituximab + low-dose steroids	Anti-proteinuric drug(s) alone	Rituximab group
5	2,63	Antiproteinuric drug(s) alone	MMF + low dose steroids	Other drugs group	MMF group
6	2,63	High-dose steroids alone	MMF + high-dose steroids	Other drugs group	MMF group
7	2,76	Cyclophosphamide + high-dose steroids	MMF + low dose steroids	Other drugs group	MMF group
8	2,99	Antiproteinuric drug(s) alone	Cyclophosphamide + high-dose steroids	Anti-proteinuric drug(s) alone	Other drugs group

MMF: Mycophenolate mofetil

Supplementary table S2 - Sensitivity analysis of renal outcomes after reassignment of patients according to treatment received within the first 3 months

	MMF-based therapy at month-3 (n = 57)	Rituximab-based therapy at month-3 (n = 28)	Other therapies at month-3 (n = 58)	p value
**PRR at 6 months**	**12/53 (22.6)**	**8/25 (32.0)**	**4/48 (8.3)**	**0.03**
CRR at 6 months	13/53 (24.5)	5/25 (20.0)	12/48 (25.0)	0.88
ORR at 6 months	25/53 (47.1)	13/25 (52.0)	16/48 (33.3)	0.22
PRR at 12 months	7/54 (13.0)	2/27 (7.4)	5/52 (9.6)	0.72
CRR at 12 months	26/54 (48.1)	16/27 (59.3)	23/52 (44.2)	0.44
ORR at 12 months	33/54 (61.1)	18/27 (66.7)	28/52 (53.8)	0.52
Class V LN relapse during follow up – n. (%)	9/55 (16.4)	4/27 (14.8)	10/57 (17.5)	0.82
Proliferative class (III or IV) renal relapse during follow up – n. (%)	5/57 (8.8)	3/28 (10.7)	5/58 (8.6)	0.95
Extra-renal relapse during follow up – n. (%)	12/57 (21.1)	5/28 (17.9)	14/58 (24.1)	0.79
Follow up duration – months median (IQR)	66.5 (38.1– 99.4)	38.0 (27.6 – 68.1)	71.2 (45.8 – 101.6)	**0.03**

CRR: Complete renal response, IQR: Interquartile range, MMF: Mycophenolate mofetil, n: Number, ORR: Overall renal response, PRR: Partial renal response

Supplementary Table S3 - Renal outcomes of patients treated with supportive care alone according to baseline proteinuria

	Proteinuria < 1 g/g (n = 13)	Proteinuria ≥ 1 g/g ; ≤ 3 g/g (n = 28)	Proteinuria > 3 g/g (n = 10)	P values
PRR at 6 months	1/13 (7.7)	4/25 (16.0)	0/8 (0)	0.70
CRR at 6 months	0/13 (0)	1/25 (4.0)	0/8 (0)	1
ORR at 6 months	1/13 (7.7)	5/25 (20.0)	0/8 (0)	0.44
PRR at 12 months	1/13 (7.7)	7/27 (25.9)	0/8 (0)	0.21
CRR at 12 months	7/13 (53.8)	11/27 (40.7)	0/8 (0)	0.03
ORR at 12 months	8/13 (61.5)	18/27 (66.7)	0/8 (0)	0.002
Use of an immunosuppressive drug for inefficacy in the first 12 months	1/13 (7.7)	5/28 (17.9)	4/10 (40)	0.14

CRR: Complete renal response, n: Number, ORR: Overall renal response, PRR: Partial renal response

Supplementary table S4 – Details of rituximab therapeutic regimens

**Rituximab therapeutic regimens**	**n=26**
375 mg/m^2 weekly during 4 weeks without maintenance	5 (19.2)
375 mg/m^2 weekly during 4 weeks with maintenance	6 (23.1)
1 gram at day 1 and 15 without maintenance	7 (26.9)
1 gram at day 1 and 15 with maintenance	5 (19.2)
Others	3 (11.5)
**n: Number**	

Supplementary table S5 – Multivariate logic regression analyses comparing renal response rates between rituximab-based regimens and other treatments groups

COMPARISON	Outcome	N	OR RTX	95% CI	P values
**RTX *vs*. MMF**	CRR+PRR	75	1.77	0.57–5.51	0.32
	CRR	75	2.33	0.76–7.08	0.14
**RTX *vs*. other IS drugs**	CRR+PRR	77	2.25	0.72–7.04	0.16
	CRR	77	2.13	0.72–6.29	0.17
**RTX *vs*. MMF and other IS drugs**	CRR+PRR	127	2.01	0.72–5.59	0.18
	CRR	127	2.21	0.84–5.86	0.11
CI: confidence interval; CRR: complete renal response; IS: immunosuppressive; MMF: mycophenolate mofetil; OR: odd ratio; PPR: partial renal response; RTX: rituximab					

Supplementary table S6 - Comparison between anti-U1RNP positive and anti-U1RNP negative patients

	Anti-U1RNP negative (n = 109)	Anti-U1RNP positive (n = 85)	p value
Age at class V diagnosis, median (IQR)	32.3 (26.1 – 39.2)	34.5 (26.5 – 40.2)	0.70
Female sex, n (%)	98/109 (89.9)	72/85 (84.7)	0.27
Months between lupus diagnosis and pure class V LN diagnosis, median (IQR)	24.0 (0 – 109.8)	11.2 (0 – 58.9)	0.18
Afro-Caribbean ethnicity, n (%)	35/109 (32)	51/85 (60)	**< 0.001**
Previous lupus kidney involvement, n (%)	29/109 (26.6)	9/85 (10.6)	**0.005**
Treatment with HCQ, n (%)	88/106 (83.2)	64/84 (76.2)	0.24
Steroids starting dose in prednisone equivalent, median (IQR)	10 (0 – 40)	30 (10 – 60)	**< 0.001**
Treatment with rituximab, n (%)	21/109 (19.3)	5/85 (5.9)	**0.007**
Treatment with MMF, n (%)	20/109 (18.3)	34/85 (40)	**0.001**
Treatment with another immunosuppressive drug, n (%)	34/109 (31.2)	27/85 (31.8)	0.93
Treatment with supportive care alone, n (%)	34/109 (31.2)	19/85 (22.4)	0.17
eGFR at diagnosis, median (IQR)	121.8 (97.7 – 132.9)	122.74 (99.3 – 135.2)	0.92
UPCr – g/g median (IQR)	2.10 (1.1 – 3.9)	2.48 (1.1 – 5.3)	0.31
Serum albumin – g/L median (IQR)	29 (21.2 – 34)	28 (21 – 32)	0.32
Hematuria, n (%)	46/97 (47.4)	30/79 (38)	0.21
C3 – mg/L median (IQR)	825 (620 – 1080)	800 (530 – 1080)	0.64
C4 – mg/L median (IQR)	140 (100 – 230)	136 (70 – 201)	0.31
Positive anti-dsDNA Abs – n. (%)	70/109 (64.2)	60/85 (70.6)	0.35
Positive anti-Smith Abs – n. (%)	11/109 (10.1)	63/85 (74.1)	**< 0.001**
Positive anti-SSA Abs – n. (%)	38/109 (34.9)	34/85 (40.0)	0.46
Positive anti-SSB Abs – n. (%)	11/109 (10.1)	8/85 (9.4)	0.87
Anti-nuclear antibody titer – median (IQR)	640 (320 – 1280)	1280 (1280 – 2560)	**< 0.001**
Arthralgia – n. (%)	37/107 (34.6)	27/85 (31.8)	0.68
Cutaneous involvement – n. (%)	32/109 (29.4)	31/85 (36.5)	0.29
Leucopenia – n. (%)	17/105 (16.2)	19/84 (22.6)	0.26
Thrombocytopenia – n. (%)	12/104 (11.5)	5/85 (5.9)	0.18
Absence of extrarenal involvement – n. (%)	35/109 (32.1à	29/85 (34/1)	0.77
IgA deposits – n. (%)	47/92 (51.1)	40/75 (53.3)	0.77

IgM deposits – n. (%)	47/92 (51.1)	41/75 (54.7)	0.64
C3 deposits – n. (%)	77/92 (83.7)	71/75 (95.7)	**0.03**
C1q deposits – n. (%)	69/92 (75)	61/75 (81.3)	0.33
Full-house deposits – n. (%)	28/92 (30.4)	29/75 (39.7)	0.26
Follow-up – years, median (IQR)	64.6 (34.6 – 107.8)	70.7 (38.2 – 95.5)	0.97

Abs: antibodies, dsDNA: Double strain deoxyribonucleic acid, eGFR: estimated glomerular filtration rate, HCQ: Hydroxychloroquine, IQR: Interquartile range, LN: Lupus nephritis, n: Number, Sm: Smith, U1RNP: U1 ribonucleoprotein, UPCR: urine protein creatinine ratio.

Supplementary table S7 - Comparison between patient with or without extrarenal involvement

	Extrarenal involvement (n = 130)	Absence of extrarenal involvement (n = 64)	p value
Age at class V diagnosis, median (IQR)	34.3 (26.1 – 40.8)	31.0 (26.6 – 37.0)	0.35
Female sex, n (%)	111/130 (85.4)	59/64 (92.2)	0.18
Months between lupus diagnosis and pure class V LN diagnosis, median (IQR)	7.8 (0 – 78.0)	49.4 (5.2 – 117.1)	**< 0.01**
Previous lupus kidney involvement, n (%)	19/130 (14.6)	19/64 (29.7)	**0.01**
Afro-caribbean ethnicity, n (%)	56/130 (43.1)	30/64 (46.9)	0.62
Treatment with HCQ, n (%)	88/106 (83.2)	64 (76.2)	0.24
Steroids starting dose in prednisone equivalent, median (IQR)	22.5 (0 – 50)	10 (0 – 35)	0.09
Treatment with rituximab, n (%)	18/130 (13.9)	8/64 (12.5)	0.80
Treatment with MMF, n (%)	37/130 (28.5)	17/64 (26.6)	0.78
Treatment with another immunosuppressive drug, n (%)	43/130 (33.1)	18/64 (28.1)	0.48
Treatment with supportive care alone, n (%)	32/130 (24.6)	21/64 (32.8)	0.23
eGFR at diagnosis, median (IQR)	121.5 (99.3 – 135.0)	123.9 (96.1 – 132.7)	0.95
UPCr – g/g median (IQR)	2.39 (1.1 – 4.5)	2.21 (1.1 – 3.4)	0.60
Serum albumin – g/L median (IQR)	27 (21 – 32)	29.3 (21 – 35)	0.09
Hematuria, n (%)	51/116 (44.0)	25/60 (41.7)	0.77
C3 – mg/L median (IQR)	815 (530 – 1070)	830 (666 – 1150)	0.37
C4 – mg/L median (IQR)	140 (80 – 210)	143 (10 – 275)	0.29
Positive anti-dsDNA Abs – n. (%)	91/130 (70.0)	39/64 (60.9)	0.21
Positive anti-Smith Abs – n. (%)	54/130 (41.5)	20/64 (31.2)	0.17
Positive anti-SSA Abs – n. (%)	54/130 (41.5)	18/64 (28.1)	0.07
Positive anti-SSB Abs – n. (%)	17/130 (13.1)	2/64 (3.1)	**0.03**
Anti-nuclear antibody titer – median (IQR)	1280 (640 – 1600)	1280 (640 – 2240)	0.91
IgA deposits – n. (%)	58/109 (53.2)	29/58 (50)	0.69
IgM deposits – n. (%)	57/109 (52.3)	31/58 (53.4)	0.89
C3 deposits – n. (%)	98/109 (89.9)	50/58 (86.2)	0.47

C1q deposits – n. (%)	87/109 (79.8)	41/58 (74.1)	0.40
Full-house deposits – n. (%)	37/109 (33.9)	20/58 (34.5)	0.94
Follow-up – years, median (IQR)	62.2 (34.7 – 95.7)	74.6 (41.4 – 112.8)	0.27
Abs: antibodies, dsDNA: Double strain deoxyribonucleic acid, eGFR: estimated glomerular filtration rate, HCQ: Hydroxychloroquine, IQR: Interquartile range, LN: Lupus nephritis, n: Number, Sm: Smith, U1RNP: U1 ribonucleoprotein, UPCR: urine protein creatinine ratio.			

Supplementary figure 1 – Sankey plot showing group allocation by number of patients

CNI: Calcineurin inhibitor, MMF: mycophenolate mofetil

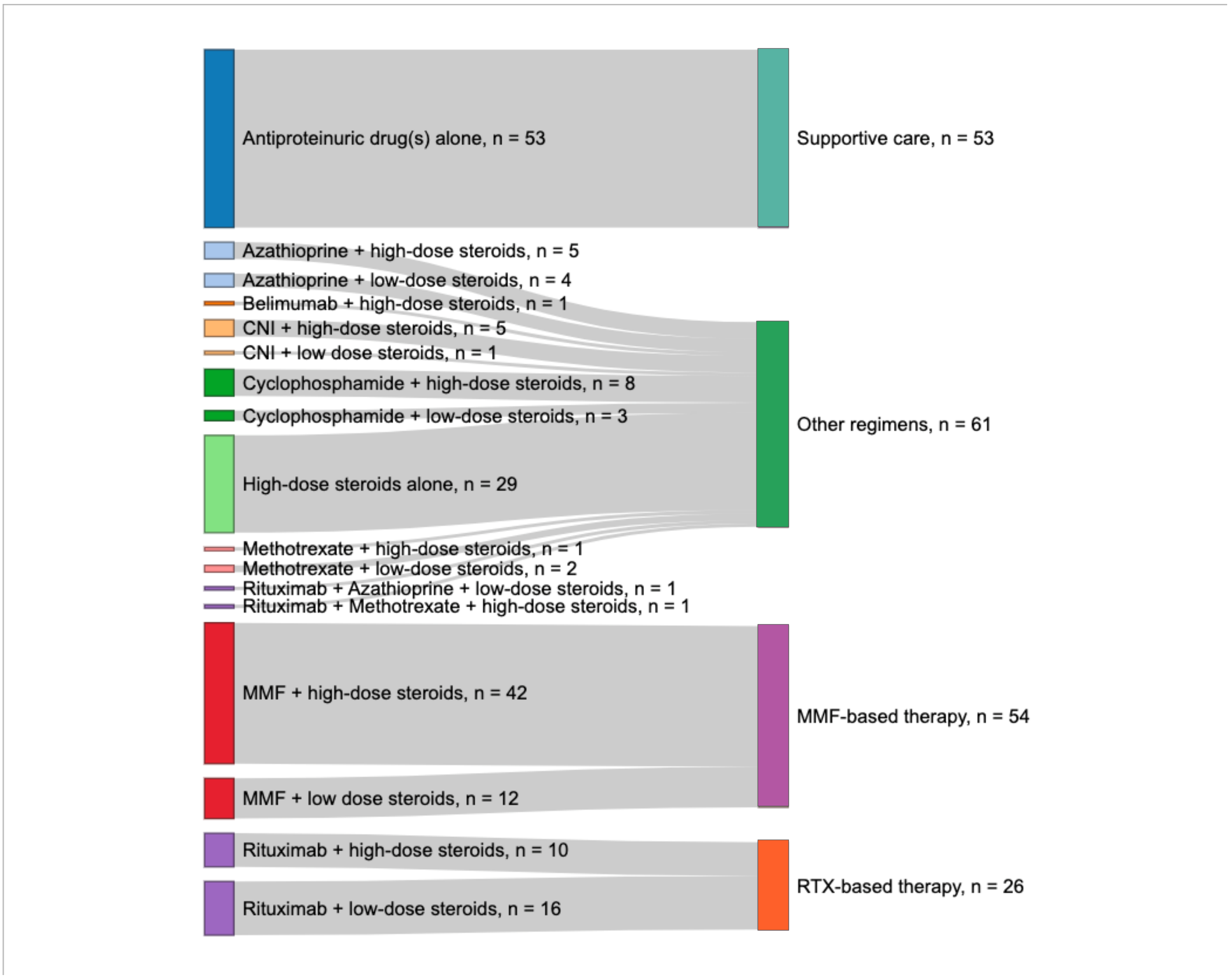

Supplementary figure 2 – Flow chart

LN: Lupus nephritis, y.o: years old

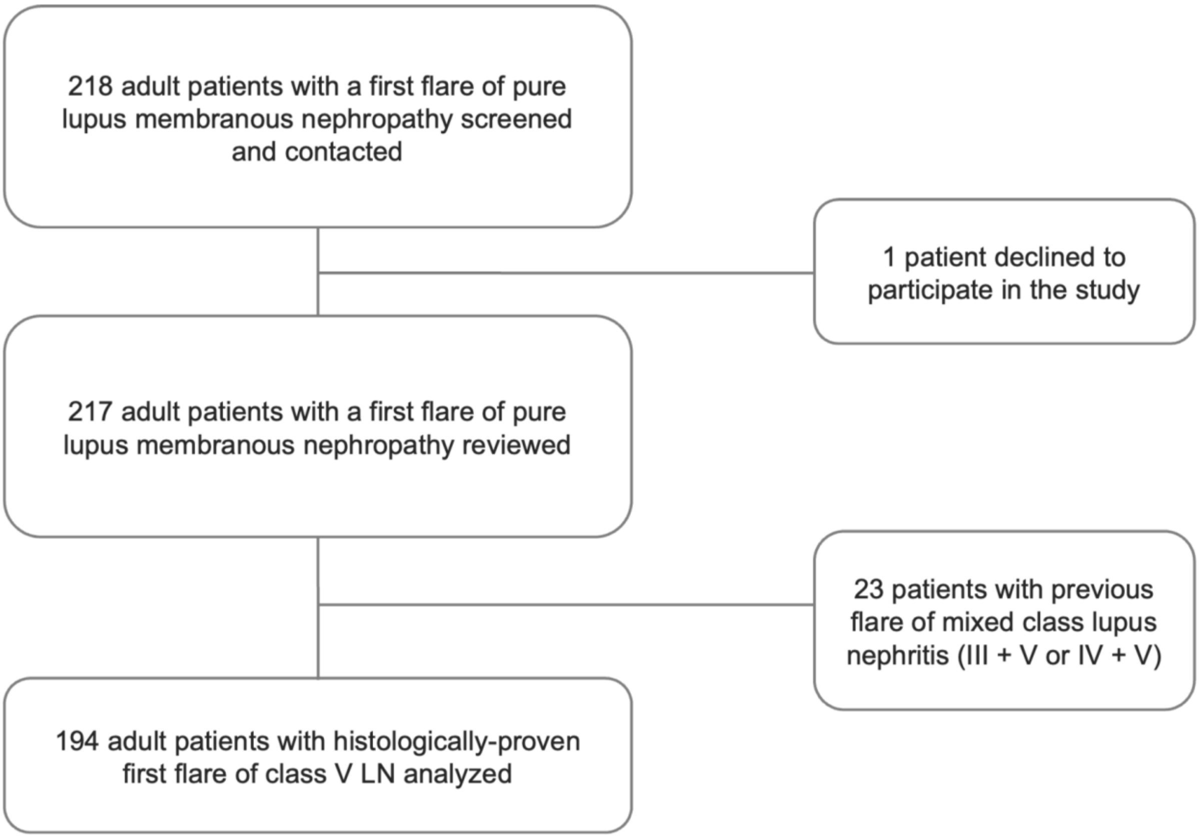

Supplement: Supplementary File (PDF) — Figure S1. Sankey plot showing group allocation by number of patients. Figure S2. Flow chart. Table S1. Characteristics of patients who switched therapy within the first 3 months after treatment initiation. Table S2. Sensitivity analysis of renal outcomes after reassignment of patients according to treatment received within the first 3 months. Table S3. Renal outcomes of patients treated with supportive care only according to baseline proteinuria. Table S4. Details of rituximab therapeutic regimens. Table S5. Multivariate logic regression analyses comparing renal response rates between rituximab-based regimens and other treatments groups. Table S6. Comparison between anti-U1RNP positive and anti-U1RNP negative patients. Table S7. Comparison between patients with or without extrarenal involvement. [file mmc1.pdf]
